# Experimental Study on the Mechanical Behaviors of Aeolian Sand Treated by Microbially Induced Calcite Precipitation (MICP) and Basalt Fiber Reinforcement (BFR)

**DOI:** 10.3390/ma16051949

**Published:** 2023-02-27

**Authors:** Jia Liu, Xi’an Li, Gang Li, Jinli Zhang

**Affiliations:** 1 School of Geological Engineering and Geomatics, Chang’an University, Xi’an 710054, China; 2 Shaanxi Key Laboratory of Safety and Durability of Concrete Structures, Xijing University, Xi’an 710123, China; 3 State Key Laboratory of Coastal and Offshore Engineering, Dalian University of Technology, Dalian 116024, China

**Keywords:** MICP, basalt fiber, aeolian sand, fiber length, fiber content

## Abstract

Aeolian sand flow is a major cause of land desertification, and it is prone to developing into a dust storm coupled with strong wind and thermal instability. The microbially induced calcite precipitation (MICP) technique can significantly improve the strength and integrity of sandy soils, whereas it easily leads to brittle destruction. To effectively inhibit land desertification, a method coupled with MICP and basalt fiberreinforcement (BFR) was put forward to enhance the strength and toughness of aeolian sand. Based on a permeability test and an unconfined compressive strength (UCS) test, the effects of initial dry density (*ρ*_d_), fiber length (*FL*), and fiber content (*FC*) on the characteristics of permeability, strength, and CaCO_3_ production were analyzed, and the consolidation mechanism of the MICP-BFR method was explored. The experiments indicated that the permeability coefficient of aeolian sand increased first, then decreased, and subsequently increased with the increase in *FC*, whereas it exhibited a tendency to decrease first and then increase with the increase in *FL*. The UCS increased with the increase in the initial dry density, while it increased first and then decreased with the increase in *FL* and *FC*. Furthermore, the UCS increased linearly with the increase in CaCO_3_ generation, and the maximum correlation coefficient reached 0.852. The CaCO_3_ crystals played the roles of providing bonding, filling, and anchoring effects, and the spatial mesh structure formed by the fibers acted as a bridge effect to enhance the strength and brittle damage of aeolian sand. The findings could supply a guideline for sand solidification in desert areas.

## 1. Introduction

Land desertification is one of the most serious ecological and environmental issues. With desertification areas accounting for one third of the land areas, nearly one billion people in more than 100 countries are affected by desertification. Currently, the common methods to control desertification include mechanical, plant, and chemical methods, whereas some drawbacks from technical, economic, and ecological perspectives still exist. The microbially induced calcite precipitation (MICP) technique is an economic, environmentally friendly, and durable method that has emerged in recent years. Calcite precipitation is induced mainly through microbial metabolic processes such as photosynthetic organisms removing carbon dioxide, sulfate-reducing bacteria, and several microorganisms participating in the nitrogen cycle [1]. Among them, the nitrogen cycle is widely used in MICP. The MICP process used bacteria with high urease activity that decomposed urea into ammonia and carbon dioxide through metabolic activities, andconverted it into ammonium ions and carbonate ions when it met water. The carbonate ions reacted with the calcium ions in the environment to form calcium carbonate precipitation [2]. Calcite crystals could bond the soil particles into a whole to achieve the aim of strength increment and seepage reduction. Gao et al. [3] found that ultraviolet radiation weakened the performance of CaCO_3_, and the porosity of aeolian sand which was treated with MICP reached 23.6% when the radiation duration reached 1046 h. Li et al. [4] concluded that the average thickness of the overlay reached 2.0–2.5 cm after 7 days of the mineralization effect, and the strength formed by staphylococcal was 1.05 times higher than that of *Bacillus*. Nikseresht et al. [5] conducted wind tunnel tests to evaluate soil loss treated by using MICP at different wind speeds and found that molasse and vinasse as growth substrates have strong consolidation to resist erosion by wind, which is mainly due to the calcium carbonate content produced and is independent of extra reformations. Zhang et al. [6] studied the feasibility of the microbially induced magnesium carbonate precipitation (MIMP) method to restrain desertification in desert districts and concluded that MIMP could counteract the weakness of conventional solidified ways, and this was in particular reference to controlled desertification in Northwest China where there are rich storage resources of magnesium. Raveh-Amit et al. [7] revealed that sufficient urea hydrolysis caused an increase in pH, which is essential to calcite deposition. The rates of biostimulation were discovered to rise with carbon source concentration and they achieved the highest levels within 3 to 6 days. Tian et al. [8,9] studied the influence of the reactant inpouring rate on cementing aeolian sand via the MICP method and concluded that the cementation sample was well distributed and the UCS varied from 4.58 to 5.03 MPa when the speed of inpouring was 0.278 mol/L/h. The CaCO_3_ crystals generated in the samples were calcite and they had polyhedral, spherical, or flower clusters in their crystal morphology. Li et al. [10] noted that with the increase in the cement concentration, calcium carbonate production and the aeolian sand density rose, the penetration rate decreased, and the UCS rose. The calcium carbonate filled the voids between the sand grains and the void volume was reduced. Based on a field test in the Ulan Buh Desert, Meng et al. [11] reported that the MICP method could conspicuously improve the bearing capacity against wind erosion through the formation of soil crusts. The optimal concentration of cementation solution was 0.2 M and the spraying amount was 4 L/m^2^. Devrani et al. [12] found a decrease in soil loss from 75.23% for unsolidification to 0% which was treated by the MICP method. The concentration of a mixture of urea and calcium chloride has an important role in soil amelioration, and the highest threshold friction velocity (TFV) was 45 km/h when the concentration was0.5 M. Dubey et al. [13] revealed a reduction of one order in the magnitude of the permeability coefficient of aeolian sand after MICP treatment, and a high UCS of around 1 MPa and the highest wind speed exceeding 55 km/h with 1 M cementation solution treatment was achieved.

With the increasing application of MICP technology, the current study faces many opportunities and challenges. Achal et al. [14] verified that the dairy industry and lactose mother liquor could be used as an alternative source of the *Sporosarcina pasteurii* (NCIM 2477) standard medium, which could support bacterial growth and urease activity. It can save more costs than using the standard medium. Xiao et al. [15] studied the thermal conductivity of biocemented silica sand samples by transient plane source method, and the experimental results showed that MICP treatment significantly improved the thermal conductivity of samples. The empirical equation of thermal conductivity of silica sand treated with MICP under different variables was established, which provided a theoretical basis for the design of energy piles in biocemented sand layers. Li et al. [16] used the MICP technique to treat the interface between calcareous sand and steel, and studied the effects of cementing materials and surface roughness on the shear properties of the interface. The results showed that MICP technology was feasible to improve the resistance of calcareous sandsteel interface in an environmentally friendly manner. However, the difference in the time, space, and material characteristics between the biological environment and the engineering system should be resolved prior to the implementation of the project. The implementation of this work required not only researchers, practitioners, and designers with the relevant knowledge background, but also good cooperation among them [17]. These are the challenges facing the application of MICP technology.

However, the contradiction between strength and toughness was a challenge to MICP-treated soils. Although the MICP-treated sand had high strength, it behaved as a typical brittle material, namely, it lost its strength instantly after damage, which had a great impact on its wind erosion resistance. To improve its strength and toughness, researchers proposed a coupled MICP and fiberreinforcement (FR) method. Wang et al. [18] found that the FR method can significantly enhance the sand’s tensile strength, peak displacement, and residual strength, and it can reduce the brittle damage at the peak strength. The tensile strength raised by 172.4% and the peak deformation increased by 158.1% when the *FC* was 0.6%. Yin et al. [19] reported that the fiber increased the colonization area and calcium carbonate deposition, which further enhanced the ductility and toughness and reduced the stiffness of the calcareous sand. Li et al. [20] revealed that fiber addition could significantly improve the solidified effect of MICP: the peak penetration resistance increased first and then decreased with the increase in the *FC*, and the optimum *FC* was 0.2%. With the increase in fiber content, Xie et al. [21] reported that the unconfined compressive strength of MICP-treated sand raised first and then reduced, amd the optimum *FC* was 0.15%. Fiber reinforcement can improve the deposition and yield of MICP, while the cementation of CaCO_3_ promotes a fiber reinforcement effect. Zhao et al. [22] conducted unconfined compressive strength and splitting tensile strength experiments to assess the influence of activated carbon-fiber felt (ACFF) on the strength of MICP-treated sand. The unconfined compressive strength and splitting tensile strength were significantly improved with small amounts of ACFF and the softnesscaused by water could be enhanced by ACFF. Qiu et al. [23] studied the strength of carbon-fiber-reinforced sand using the UCS test and concluded that UCS rose first and then reduced with the increase in *FC*. The optimum *FC* of silica and calcareous sand was 0.2% and 0.1%, respectively. Li et al. [24] revealed remarkable enhancements in shear strength, malleability, and failure strain when fibers were added to MICP-treated sand. The unconfined compressive strength rose gradually with the increase in *FC* when the *FC* was less than 0.3%, and the optimal *FC* ranged from 0.2% to 0.3%. Hao et al. [25] pointed out that MICP modification could improve the crack-resistant properties and energy absorption capacity of fiber-reinforced cementitious composite beam samples by 58% and 69.3%, respectively. Lei et al. [26] reported that the ductility, bridging role, and CaCO_3_ production of biocemented calcareous sand were significantly improved in the following order of carbon fiber, basalt fiber, and glass fiber. The unconfined compressive strength, tensile strength, and CaCO_3_ production rose with the increase in *FC*, and the optimal *FC* was found to be 1%. Imran et al. [27] discovered that fiber content more remarkably affected the ductility, toughness, and brittleness of the MICP-treated sand than *FL*, and the optimum *FC* and *FL* were 3% and 15 mm, respectively. Fang et al. [28] reported that *FC* has an influence on the engineering characteristics of MICP-treated coral sand which is more remarkable than *FL*, and the optimum *FC* and *FL* were 0.2% and 9 or 12 mm, respectively. Wen et al. [29] concluded that fiber addition in MICP-treated bio-beams could improve their ductility, and the optimal *FC* was about 0.3%. Under the optimum fiber content, the post-peak flexural strength loss was prevented, and the ductility of the bio-beams was improved. Spencer et al. [30] found that the addition of jute fibers in the MICP-treated sand was beneficial, leading to irritation of bacterial activity and maintenance of the MICP process during a 12-d treatment. Choi et al. [31,32] concluded that the splitting tensile strength and secant elastic modulus raised with the increase in CaCO_3_ content or *FC*, and the failure strain and post-failure splitting tensile strength were improved by polyvinyl acetate fiber merging with MICP treatment. In addition, fibers facilitate the MICP process by bridging the voids between sand grains. Yao et al. [33] indicated that wool fibers supplied nucleation sites for CaCO_3_ and reduced porosity, and the generated calcite was distributed in the fiber-bridging microstructure uniformly, which was significantly enhanced by the MICP process.

In conclusion, the combination of fiber reinforcement and MICP technology (MICP–FR) has applicable value for improving the strength and toughness of soil. The present research results show that MICP–FR technology is mainly used to solidify calcareous sand, standard sand, coral sand, etc., and there are relatively few studies on aeolian sand. Therefore, in this paper, MICP and basalt fiberreinforcement (BFR) methods were coupled to solidify aeolian sand to explore its mechanical properties and applicability. The mechanical properties of solidified aeolian sand were investigated based on permeability and UCS tests, and the consolidation mechanism of the MICP–BFR method was explored through a scanning electron microscope (SEM) test. The findings could supply a guideline for the study of the solidification of aeolian sand and sand control in desert areas.

## 2. Materials and Methods

### 2.1. Experimental Materials

**Figure 1:**
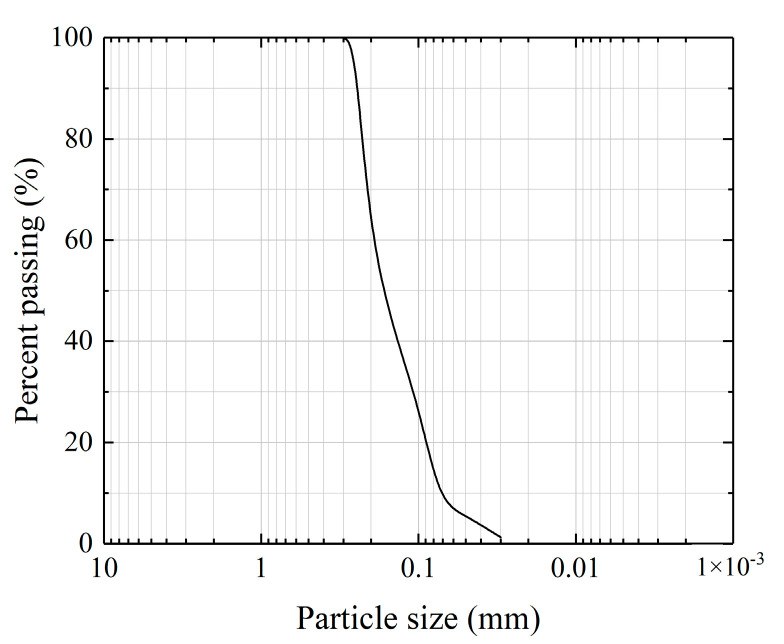
Particle size distribution curve of the aeolian sand.

Aeolian sand was collected from the Ulan Buh Desert in Inner Mongolia, China, and the curvature coefficient and uniformity coefficient were 0.88 and 2.58, respectively. Based on the standard for soil test method (GB/T 50123-2019), the sand was defined as poorly graded fine sand, and Table 1 lists the physical properties of the aeolian sand. Figure 1 is the particle size distribution curve of the aeolian sand.

*Bacillus pasteurii* (ATCC 11859) was used in the test and it was purchased from the Shanghai Bioresource Collection Center, Shanghai, China. CASO AGAR + urea (20 g/L) was adopted as the culture medium for the bacteria, which had the following contents: tryptone (15 g), peptone (5 g), sodium chloride (5 g), agar powder (20 g), purified water (900 mL), and 20% urea (100 mL). During the test, the pH of the culture medium was adjusted to 9.0, and then, the growth medium was sterilized under 121 °C for 15 min. After cooling to 60 °C, it was added to 100 mL of 20% urea that had been filtrated and sterilized. Subsequently, 1 mL of bacteria solution concentration (OD_600_ = 1.0) was added to the growth medium, and it was cultured in a shaker at 32 °C with a shaking frequency of 170 rpm. Cultivation was stopped when the OD_600_ reached 1.5, and the culture medium was placed into a refrigerator at a temperature of 4 °C for storage. The cementation solution in the test was a mixture of CaCl_2_ and urea with an equal concentration and volume.

Basalt fiber is a natural fiber with high strength, corrosion prevention, and hightemperature resistance properties. It is a green and environmentally friendly material with little environmental pollution. The basalt fibers selected for the experiment were smooth, bunched and approximately golden. They were purchased from the same company as Kou et al. [34] and their performance is shown in Table 2.

### 2.2. Sample Preparation

**Figure 2:**
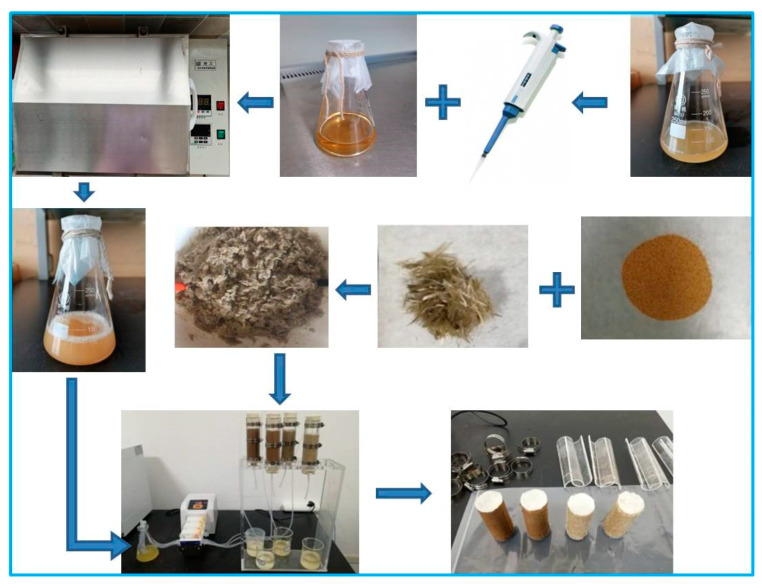
The process of sample preparation.

A Plexiglas tube with an inner diameter of 39.1 mm and a height of 150 mm was adopted as the specimen mold. It was opened in half to facilitate the removal of the specimen and fixed with a stainless-steel clamp. The aeolian sand samples were 39.1 mm in diameter and 80 mm in height. In order to prevent leakage during the grouting process, a thin plastic sheet was placed to cover the sample. During the test, the aeolian sand was first passed through a 0.5 mm sieve. Then, the basalt fibers with a fixed fiber length (*FL*) and fiber content (*FC*) were disassembled into filaments and sunk in water to facilitate dispersion. Subsequently, the fibers were mixed evenly with the aeolian sand and loaded into the mold according to the preset dry density (*ρ*_d_). The samples were prepared in four layers and ascraping process was carried out between the layers. Figure 2 exhibits the process of sample preparation. Three layers of filter paper were laid under the sample before loading, and a layer of filter paper was placed on top of the sample after loading. The upper and lower areas of the mold were blocked with rubber plugs with holes. During the test, the bacterial solution and the cementation solution were injected into the tube with a peristaltic pump at a rate of 2 mL/min.

### 2.3. Test Methodology

Table 3 shows the test scheme, where the cementation number refers to the grouted number of the cementation solution. The test used a repeated staged injection method, and the bacterial solution was grouted and stood for 3 h. Then, the cement liquid was poured four times, at an interval of 16 h. After grouting the bacterial solution for the last time, the cementation solution was grouted twice. After the solution was completely perfused, the samples were flushed with pure water at the same rate three times to terminate the MICP reaction and eliminate the by-products in the MICP process. The permeability test and UCS test were conducted on solidified aeolian sand. The permeability test used the variable head method to measure the permeability coefficient, and the UCS test used a soil triaxial instrument to measure the UCS. The waterhead of the permeability test was 1.5 m. After the tube was filled with water to the required height, the height and time of three different initial heads were measured, and the average value of the three calculated results was taken as the permeability coefficient. The UCS test was loaded at a strain rate of 1.0%/min, and the experiment was paused when the peak strength appeared or the axial strain reached 15%. All of the tests were repeated to eliminate any errors.

## 3. Results and Discussion

### 3.1. Permeability Characteristics of Solidified Aeolian Sand

**Figure 3:**
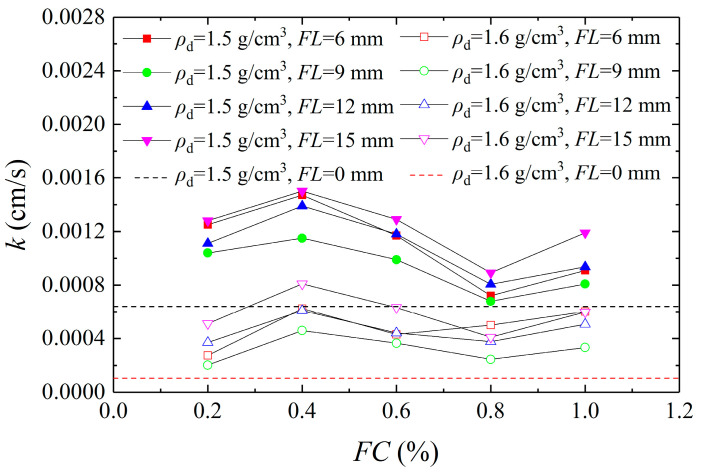
Curves of permeability coefficient versus fiber content.

For the sake of analysis of the influence of the MICP–BFR method on the permeability characteristics of aeolian sand, Figure 3 shows the curves of the permeability coefficient versus the fiber content. The dotted lines in the figure indicate the permeability of aeolian sand solidified with MICP. It can be seen that the permeability of the aeolian sand was significantly improved after the addition of basalt fiber. When the initial dry densities of the samples were 1.5 g/cm^3^ and 1.6 g/cm^3^, the permeability coefficient first increased, then decreased, and finally increased with the increase in *FC*. When the *FC* was 0.2–0.4%, the permeability coefficient increased. When the *FC* was small, the fiber was distributed among the sand particles, forming a seepage channel in the sample, leading to an increase in the permeability coefficient. When the *FC* was 0.4–0.8%, the permeability coefficient decreased with the increase in *FC*. When the *FC* increased, the fiber distribution became tighter and overlapped, which damaged the integrity of the sample, blocked the drainage path of the sample, and led to a decrease in the permeability coefficient. When the *FC* was 0.8–1.0%, the permeability coefficient increased gradually. When excessive fibers were added, the sand particles were distributed more dispersedly, and the pores formed by the fibers in the sample were dominant, forming new seepage channels and increasing the permeability coefficient. This is inconsistent with the research results of Choi et al. [31]. Possible reasons for the differences are the different perfusion methods and the amount of solution, which are related to the production of calcium carbonate, which affects the permeability of the sample.

**Figure 4:**
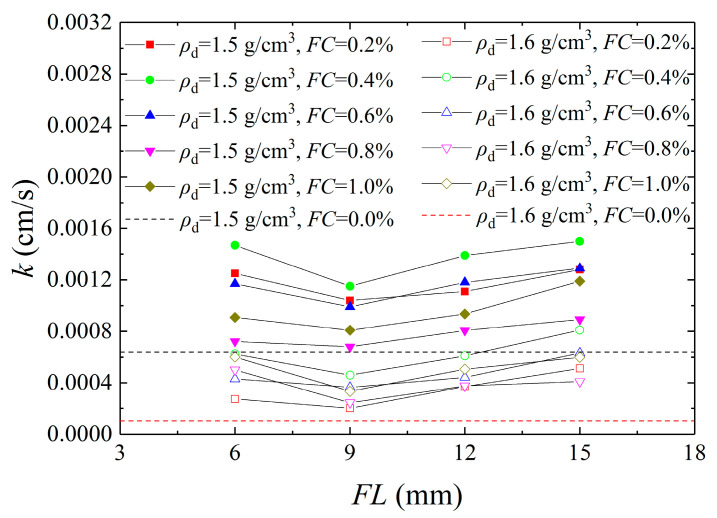
Curves of permeability coefficient versus fiber length.

Figure 4 shows the relationship between the permeability coefficient and fiber length. It can be seen that the permeability coefficient reduced first and then rose with an increase in *FL*. When the *FL* was less than 9 mm, the permeability coefficient decreased, and when the *FL* was more than 9 mm, the permeability coefficient increased gradually. When the *FL* was shorter, the fibers became nucleation sites for bacteria, and CaCO_3_ crystals adhered to the fiber surfaces and filled the pores between the sand grains, leading to a reduction in the permeability coefficient. With the increase in *FL*, the fibers determined the directional distribution of the pores and formed penetration paths inside the sample, increasing the permeability coefficient. The conclusions that come from the experimental results are that the permeability coefficient of solidified aeolian sand was lowest when the *FL* and *FC* were 9 mm and 0.8%, respectively.

### 3.2. Effect of Initial Dry Density on the UCS of Solidified Aeolian Sand

**Figure 5:**
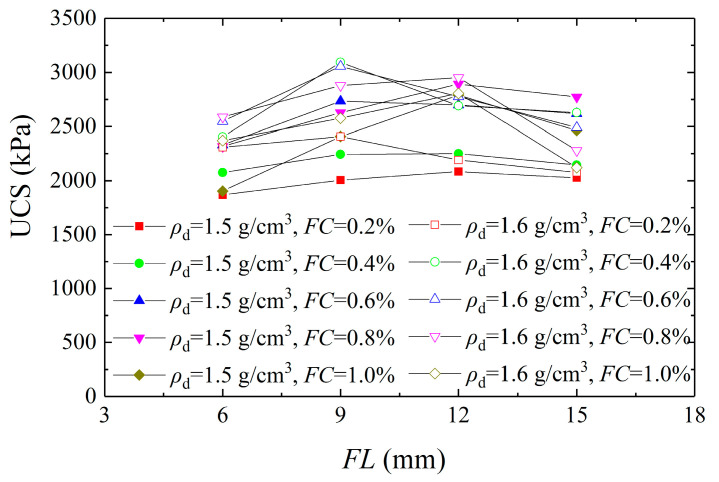
Curves of unconfined compressive strength versus fiber length.

UCS is a significant parameter that reflects the strength properties of soil. To establish the effect of initial dry density on the UCS of solidified aeolian sand, Figure 5 shows the curves of unconfined compressive strength versus fiber length. It can be observed that the UCS increased first and then decreased with the increase in *FL*, and peak strength appeared with 9 mm *FL*. With the increase in *FC*, the UCS increased first and then decreased, and peak strength appeared with 0.8% *FC*. According to the comparison of the test results, it can be seen that the UCS of aeolian sand with a dry density of 1.5 g/cm^3^ was significantly lower than that of 1.6 g/cm^3^, thus indicating that the UCS increased with the increase in dry density, which is consistent with the conclusions of Fang et al. [28]. The primary cause is that the pores among sand grains gradually decreased as the dry density increased, and the CaCO_3_ crystals effectively filled the pores and cemented the sand particles, thereby improving the strength of aeolian sand. In addition, the CaCO_3_ crystals cemented the fibers and sand grains together, and the fibers played a bridging role, and shared the external loading with the sand particles. Owing to the high tensile strength of the fibers, the strength of solidified aeolian sand can be further improved when subjected to external loading.

### 3.3. Effect of Fiber Content on the UCS of Solidified Aeolian Sand

**Figure 6:**
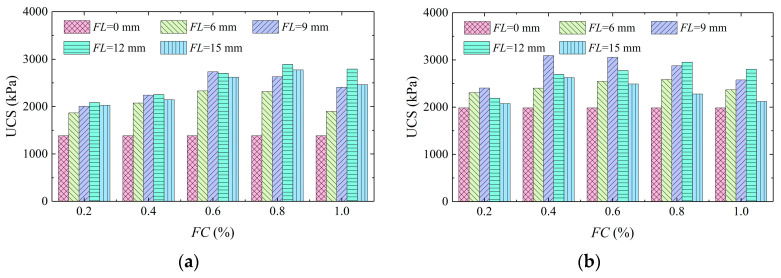
Histogram of unconfined compressive strength versus fiber content (**a**) *ρ*_d_ = 1.5 g/cm^3^; and (**b**) *ρ*_d_ = 1.6 g/cm^3^.

Figure 6 exhibitsa histogram of UCS versus fiber content. It can be seen that the UCS of aeolian sand was notably increased by the addition of fibers, and the UCS increased first and then reduced with the increase in *FC*, which is consistent with the conclusions of Qiu et al. [23]. When the dry density of the sample was 1.5 g/cm^3^, the UCS of the unreinforced sample was 1382.22 kPa, and the minimum UCS of the reinforced sample was 1869.18 kPa, indicating that the addition of fiber can increase the strength of the sample. The UCS was higher when the *FC* was 0.6% and 0.8%. When the dry density of the sample was 1.6 g/cm^3^, the UCS of the unreinforced sample was 1983.91 kPa, which was lower than the minimum UCS of the reinforced sample which was 2076.92 kPa. The UCS was larger when the *FC* was 0.4% and 0.6%. The main reason for this is that the basalt fibers had a charge, and it was easy for them to adsorb each other. When the *FC* was higher, more fibers came into contact to form clumps, leading to the nonuniform distribution of fibers in the samples. In addition, the pores between sand particles were occupied by fibers, which restrained the flow of bacteria, leading to uneven distribution and inhibiting the volume of CaCO_3_ crystals, which weakened the solidified effect of aeolian sand [35].

### 3.4. Effect of Fiber Length on the UCS of Solidified Aeolian Sand

**Figure 7:**
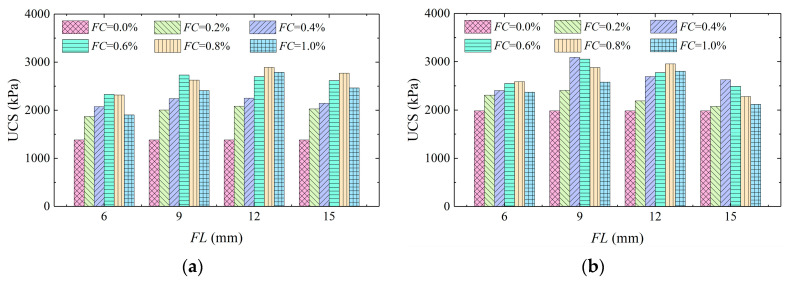
Histogram of unconfined compressive strength versus fiber length (**a**) *ρ*_d_ = 1.5 g/cm^3^; and (**b**) *ρ*_d_ = 1.6 g/cm^3^.

Figure 7 displays a histogram of UCS versus fiber length. It can be seen that the UCS increased first and then decreased with the increase in *FL*, thus indicating that fiber reinforcement can significantly improve the strength of aeolian sand, and an optimal *FL* was present. When the dry densities of the samples were 1.5 g/cm^3^ and 1.6 g/cm^3^, the UCS was higher at an *FL* of 12 mm and 9 mm, respectively. A single tensile effect and interspace reticulate structure of basalt fibers could increase the strength and toughness of aeolian sand meanwhile. The long fibers had a large contact area with the soil particles and CaCO_3_ crystals and could form a wider transmission system. Therefore, the overall performance was better when the samples were subjected to compressive loading. However, the fibers experienced a bending and overlapping phenomenon when the *FL* was sufficient, thus leading to inhibition of the transmission effect and the entire mechanical performance [35]. On the whole, when the dry density of the sample was 1.5 g/cm^3^, the UCS was higher at 12 mm *FL* and 0.8% *FC*. When the dry density was 1.6 g/cm^3^, the reinforcement effect was superior at 9 mm *FL* and 0.4% *FC*.

### 3.5. Relationship between UCS and CaCO_3_ Generation

**Figure 8:**
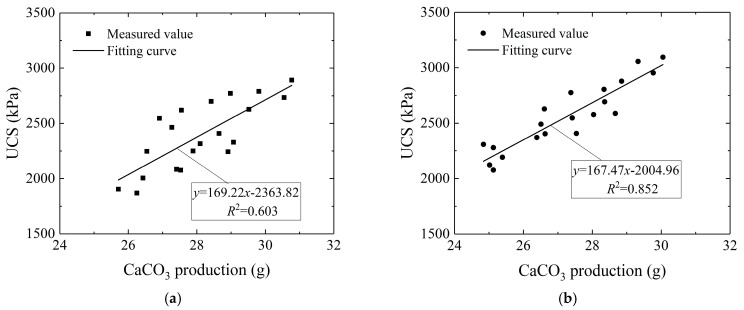
Curves of unconfined compressive strength versus CaCO_3_ content (**a**) *ρ*_d_ = 1.5 g/cm^3^; and (**b**) *ρ*_d_ = 1.6 g/cm^3^.

The generated CaCO_3_ reflected the merit of the MICP process and affected the UCS of aeolian sand. In order to research the effect of CaCO_3_ production on the permeability and strength properties of aeolian sand, the relationships between the permeability coefficient, UCS, and CaCO_3_ content were analyzed. The regression analysis results indicated that the correlation between the permeability coefficient and CaCO_3_ content was not obvious, so no further analysis will be carried out. Figure 8 exhibits the curves of UCS versus CaCO_3_ generation. The UCS of aeolian sand increased linearly with the increase in CaCO_3_ generation [31], and the correlation coefficient reached 0.852 at the dry density of 1.6 g/cm^3^, which was significantly higher than that of 1.5 g/cm^3^. During the MICP process, CaCO_3_ crystals filled the voids and cemented sand grains, leading to an increase in the cohesion between the sand particles. In the meantime, the CaCO_3_ crystals demonstrated an anchoring effect on the fibers, and the fibers interwove to form a mesh structure and bonded with the sand particles as a whole. The cement effect of the CaCO_3_ crystals and the tensile effect of the fibers were well demonstrated, leading to an improvement in the strength of aeolian sand.

### 3.6. Consolidation Mechanism of MICP-BFR Treated Aeolian Sand

**Figure 9:**
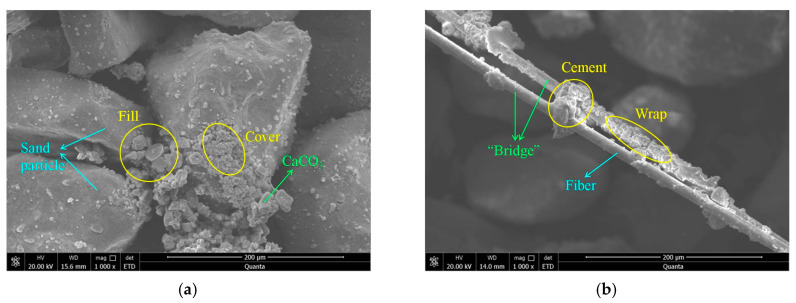

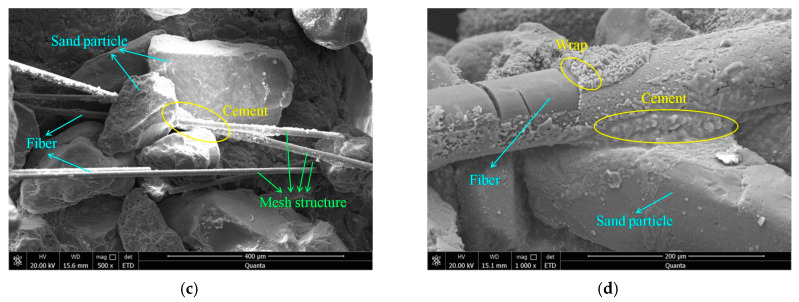
SEM images of solidified aeolian sand (**a**) CaCO_3_ distribution; (**b**) bridging effect; (**c**) mesh structure; and (**d**) composite material.

In order to reveal the consolidation mechanism of the MICP–BFR method, SEM tests were conducted on the sample of aeolian sand, and Figure 9 shows the SEM images of solidified aeolian sand. As represented in Figure 9a, it can be seen that the CaCO_3_ crystals were mainly distributed on the sand surface and filled the voids between the sand grains, which played the roles of cementing and filling. The microscopic morphology of CaCO_3_ crystals was prismatic, indicating that the CaCO_3_ crystals were mainly calcite, and the stability and deposition rate were significantly superior to aragonite and vaterite [36]. As shown in Figure 9b, it can be seen that the CaCO_3_ crystals were primarily deposited on the fiber surfaces and between the fibers, and they demonstrated an anchoring effect. The bridging effect of the fibers can enhance the strength and toughness when the sample suffered from compressive loading. As shown in Figure 9c, the fibers interwove to form a spatial mesh structure, which limited the deformation of the sand particles to a certain extent. When the samples encountered external loading, the fibers on the shear band generated tensile stress, which further compensated for the strength losses. However, the reinforcement effect was insignificant due to part of the fibers experiencing cohesion failure. As seen in Figure 9d, the CaCO_3_ crystals attached to the fiber surfaces, resulting in an improvement of the fiber roughness and an increase in the contact area between the fibers and sand grains. A composite was formed by the sand particles, CaCO_3_ crystals, and fibers, which resists deformation according to mutual friction [37]. Understanding the mechanism of MICP–BFR-treated aeolian sand could help the research between MICP–BFR and other disciplines, and apply it to newer fields [38,39,40].

## 4. Conclusions

Based on the permeability test and UCS test, the mechanical properties of cemented aeolian sand were investigated, and the consolidation mechanism of the MICP–BFR method was explored according to the SEM test. The main results are as follows:

(1) With the increase in *FC*, the permeability coefficient presented three stages of increase-decrease-increase. With the increase in *FL*, it first decreased and then increased. When the *FC* was 0.2–0.4%, the permeability coefficient increased gradually and then decreased at 0.4–0.8%. When the *FC* was 0.8–1.0%, the permeability coefficient showed an increasing trend again. When the *FL* was less than 9 mm, the permeability coefficient decreased and then increased as the *FL* was greater than 9 mm. The permeability coefficient was lower at 0.8% *FC* and 9 mm *FL*, respectively, whereas it was higher at 0.4% *FC* and 15 mm *FL*, respectively.

(2) The UCS of aeolian sand rose with the increase in dry density, and the strength of the samples with a dry density of 1.5 g/cm^3^ was significantly lower than that of 1.6 g/cm^3^.

(3) The UCS of aeolian sand rose first and then decreased with the increase in *FL* and *FC*. When the dry density was 1.5 g/cm^3^, the optimum reinforcement condition was 12 mm *FL* and 0.8% *FC*, while it was 9 mm *FL* and 0.4% *FC* when the dry density was 1.6 g/cm^3^.

(4) There was no obvious correlation between the permeability coefficient and CaCO_3_ production, while the UCS increased linearly with the increase in CaCO_3_ yield. When the dry density was 1.6 g/cm^3^, the correlation coefficient reached 0.852.

(5) The CaCO_3_ crystals demonstrated cementing, filling, and anchoring effects. The interspace reticulate structure shaped by the fibers played a bridging effect to restrain displacement, and the high tensile strength of the fibers can be compensated by the brittle damage of the samples.

In this paper, a method of combining basalt fiber and microbially induced calcite precipitation technology to solidify aeolian sand is proposed. The experimental results show that this method improves the strength of microbial solidified sand to some extent. However, limitations exist in the research. From the test results, the influence of this method on the permeability coefficient is relatively complex. Verifying this through theory requires further research. It has been suggested that the content and length of the fibers should be smaller (FC < 0.6%, and FL < 9 mm) in future relevant research.

## Figures and Tables

**Table 1:** Physical properties of aeolian sand.

*ρ*_dmax_ (g/cm^3^)	*ρ*_dmin_ (g/cm^3^)	*d*_10_ (mm)	*d*_30_ (mm)	*d*_60_ (mm)	*C*_u_	*C*_c_
1.85	1.47	0.073	0.109	0.187	2.58	0.88

**Table 2:** Physical and mechanical properties of basalt fiber [34].

Diameter (μm)	Tensile Strength (MPa)	Elastic Modulus (GPa)	Density (g/cm^3^)	Elongation at Fracture (%)
10	3500–4500	100	2.65	2.2

**Table 3:** Test scheme.

*ρ*_d_ (g/cm^3^)	Cementation Number	Fiber Length (mm)	Fiber Content (%)
1.5	18	6	0.2, 0.4, 0.6, 0.8, 1.0
	18	9	0.2, 0.4, 0.6, 0.8, 1.0
	18	12	0.2, 0.4, 0.6, 0.8, 1.0
	18	15	0.2, 0.4, 0.6, 0.8, 1.0
1.6	18	6	0.2, 0.4, 0.6, 0.8, 1.0
	18	9	0.2, 0.4, 0.6, 0.8, 1.0
	18	12	0.2, 0.4, 0.6, 0.8, 1.0
	18	15	0.2, 0.4, 0.6, 0.8, 1.0

## Data Availability

Not applicable.
